# Twelve-month pelvic pain after ovarian-preserving laparoscopic hysterectomy: Secondary analysis of a randomized trial of hyaluronic acid gel

**DOI:** 10.1016/j.eurox.2026.100490

**Published:** 2026-09-12

**Authors:** Maryam Hashemi, Fatemeh Radmanesh, Safoura Rouholamin, Mohammadreza Elhaie

**Affiliations:** a Department of Obstetrics and Gynecology, School of Medicine, Isfahan University of Medical Sciences, Isfahan, Iran; b Department of Radiology, School of Medicine, Isfahan University of Medical Sciences, Isfahan, Iran

**Keywords:** Hysterectomy, Laparoscopy, Pelvic Pain, Tissue Adhesions, Hyaluronic Acid, Randomized Controlled Trial

## Abstract

**Background:**

Postoperative pelvic pain is a clinically relevant outcome after laparoscopic hysterectomy with ovarian preservation. Hyaluronic acid barriers can reduce postoperative adhesion formation, but whether this translates into lower long-term patient-reported pelvic pain remains uncertain.

**Methods:**

This secondary analysis of a patient- and outcome-assessor-blinded randomized controlled trial included 180 women undergoing total laparoscopic hysterectomy with preservation of one or both ovaries for benign indications. Participants were randomized 1:1 to receive intraoperative hyaluronic acid gel or no barrier. The primary outcome evaluated in this secondary analysis was patient-reported pelvic pain at 12 months using the Numerical Rating Scale (NRS). Analgesic use, postoperative complications, cystic lesions, and exploratory sonographic findings suggestive of adhesions were also evaluated. Multivariable logistic regression was treated as exploratory.

**Results:**

All participants completed 12-month pain follow-up. Median pain scores were 0 [IQR, 0–2] with gel and 2 [IQR, 0–4] without gel (P < 0.001). Pain (NRS ≥1) was reported by 44/90 (48.9%) participants in the gel group versus 66/90 (73.3%) in the control group, corresponding to an absolute risk difference of −24.4 %age points (95% CI, −38.2 to −10.7) and a relative risk of 0.67 (95% CI, 0.52–0.85). Analgesic use was lower with gel: 25.6% versus 47.8% (P = 0.003). Exploratory sonographic findings suggestive of adhesions and cystic lesions were less frequent in the gel group.

**Conclusion:**

In this secondary analysis, intraoperative hyaluronic acid gel application was associated with lower patient-reported pelvic pain at 12 months after ovarian-preserving laparoscopic hysterectomy. Because the parent trial was not powered specifically for long-term pain and baseline pain trajectories were not prospectively quantified, these findings should be considered hypothesis-generating and require confirmation in larger multicentre trials with prospectively specified patient-reported outcomes.

## Introduction

Hysterectomy remains one of the most commonly performed gynecologic surgeries worldwide for benign indications such as uterine fibroids, abnormal uterine bleeding, and adenomyosis [[1], [2], [3], [4], [5], [6]]. Although most women report satisfaction with the procedure, a clinically important minority experience persistent or new-onset pelvic pain. Reported rates vary substantially according to the population, baseline pain status, definition of persistent pain, and duration of follow-up [1,5,7]. Persistent postoperative pelvic pain should not be considered synonymous with chronic pelvic pain, adhesion-related pain, residual ovary syndrome, or ovarian remnant syndrome; these are distinct clinical concepts with overlapping but non-equivalent presentations.

Ovarian conservation at the time of hysterectomy is frequently performed in premenopausal women to preserve hormonal function and avoid immediate menopausal symptoms. Residual ovary syndrome refers to symptoms arising from intentionally retained ovarian tissue after hysterectomy, whereas ovarian remnant syndrome generally refers to symptomatic residual ovarian tissue after an intended oophorectomy. Neither condition is synonymous with persistent postoperative pelvic pain. In the context of the present study, postoperative adhesions provide the principal biological rationale for use of an anti-adhesion barrier. Adhesions may restrict organ mobility and contribute to visceral or traction-related pain in selected patients, although the relationship between adhesion burden and pain severity is not linear and pain is multifactorial.

Laparoscopic surgery reduces, but does not eliminate, the risk of postoperative adhesion formation. Anti-adhesion barriers such as hyaluronic acid-based gels act as temporary physical separators during the early postoperative period. Contemporary systematic-review evidence supports a reduction in postoperative adhesion formation with hyaluronic-acid products in gynecologic surgery, including reductions in moderate-to-severe adhesions and adhesion sites. However, most studies evaluate anatomical adhesion outcomes rather than long-term patient-reported pain, and evidence that preventing adhesions necessarily prevents chronic postsurgical pain remains limited. Persistent postsurgical pain may also reflect preoperative pain phenotype, central sensitization and nociplastic mechanisms, psychological distress, acute postoperative pain, inflammatory complications, surgical duration, peripheral or neuropathic mechanisms, and perioperative analgesic exposure. Therefore, any pathway from hyaluronic acid to reduced 12-month pain through adhesion prevention remains biologically plausible but unproven.

Given the high prevalence of hysterectomy, the clinical importance of persistent postoperative pain, and the limited long-term randomized evidence linking anti-adhesion barriers with patient-reported outcomes, further investigation is warranted. The present manuscript reports a secondary analysis focused on 12-month patient-reported pelvic pain and related postoperative outcomes.

## Methods

### Study design

This study was a randomized, patient- and outcome-assessor-blinded, parallel-group clinical trial conducted at BlindedHospitals, affiliated with Blinded University of Medical Sciences, from 2024 to 2025. This manuscript presents a secondary analysis focused on 12-month patient-reported pelvic pain and related postoperative outcomes. The study protocol was approved by the Ethics Committee of Blinded University of Medical Sciences and registered in the Blindedian Registry of Clinical Trials. All procedures were performed in accordance with the Declaration of Helsinki. Written informed consent was obtained from every participant prior to enrollment. The available study documentation does not permit independent verification of all elements of the parent trial endpoint hierarchy or the timing of finalization of the statistical analysis plan relative to unblinding; these issues are acknowledged as limitations.

### Participants

Women aged 30–50 years who were scheduled for total laparoscopic hysterectomy (TLH) with preservation of one or both ovaries for benign gynecological indications, such as uterine myomas or abnormal uterine bleeding, were eligible. Patients were excluded if they had a history of endometriosis, fibromyalgia (diagnosed by the Fibromyalgia Survey Questionnaire), pre-existing chronic pelvic pain, abdominal malignancy, pelvic organ prolapse, current or recent corticosteroid use, urinary incontinence, or a diagnosed psychiatric disorder confirmed by a psychiatrist. Women undergoing hysterectomy for malignant or premalignant lesions requiring chemotherapy or radiotherapy were also excluded. Participation was voluntary, and patients could withdraw consent at any stage of the study.

### Randomization and blinding

Eligible patients were randomly allocated in a 1:1 ratio to either the intervention group (anti-adhesion barrier) or the control group (no barrier) using computer-generated randomization performed with Random Allocation Software by an independent statistician not involved in patient care. Allocation was concealed using sequentially numbered opaque sealed envelopes. The trial was patient- and outcome-assessor-blinded. Because the intervention involved intraoperative application of the gel, the operating surgeons could not be blinded; however, they had no role in postoperative data collection, pain assessment, outcome adjudication, or statistical analysis. Patients and assessors remained unaware of group assignment throughout the study.

### Surgical procedure and intervention

All operations were performed by experienced gynecologic laparoscopists using a standardized technique for total laparoscopic hysterectomy with ovarian conservation. The type of anesthesia (general or spinal) was chosen at the discretion of the anesthesiologist. Intraoperative data, including operative time, estimated blood loss, and any immediate complications such as bowel or bladder injury and the need for blood transfusion, were prospectively recorded. Postoperative management followed standard institutional protocols in both groups.

### Outcome measures

For the present secondary analysis, the main patient-reported outcome was pelvic pain intensity at 12 months postoperatively, assessed using the Numerical Rating Scale (NRS), where 0 indicates no pain and 10 indicates the worst pain imaginable. For the principal binary analysis, any reported pain was defined as NRS ≥ 1. This threshold captures even mild discomfort and should not be interpreted as a validated definition of clinically significant chronic postsurgical pain. A clinically preferable NRS ≥ 3 analysis could not be reconstructed for this revision because participant-level NRS values and NRS ≥ 3 event counts are not available in the aggregate dataset; accordingly, no unsupported NRS ≥ 3 estimates are reported. Patients were asked to report the average severity of pelvic pain, cramping, or discomfort experienced during the preceding year, so recall bias is possible. Cyst development and exploratory sonographic findings suggestive of adhesions were assessed by transvaginal ultrasound at the 12-month visit by a radiologist blinded to group allocation. Because postoperative adhesions cannot be directly or definitively diagnosed or graded by ultrasound, these findings were considered indirect exploratory indicators rather than objective adhesion grading. No formal inter-observer reliability assessment was performed. Cysts were defined as ovarian or pelvic cystic lesions > 1 cm. Not all patients underwent ultrasound, and missing imaging data limit conclusions regarding adhesion-related findings. Baseline characteristics such as age, body mass index, comorbidities, parity, type of previous deliveries, indication for hysterectomy, type of anesthesia, and operative details were also collected; quantitative baseline pelvic pain was not available.

### Follow-up

Follow-up procedures were designed to maximise retention. All participants received a detailed schedule of follow-up contacts at discharge. A dedicated research coordinator made repeated telephone attempts on different days and at different times, including evenings and weekends, with prompt text-message reminders and telephone assessment when necessary. Thus, follow-up was actively maintained through the 12-month assessment; however, the pain measure itself was obtained at the 12-month assessment and required retrospective reporting of the preceding year rather than serial pain measurements at predefined intervals.

### Sample size calculation

Sample size was calculated for the parent trial based on adhesion-related complications leading to hospital admission (expected 33% vs 64%, α = 0.05, power = 98%, 10% attrition), resulting in 90 patients per group. The parent trial was therefore not specifically powered on the basis of the 12-month patient-reported pain outcome examined in this secondary analysis.

### Statistical analysis

Data were analyzed using SPSS software. Continuous variables with normal distribution were compared between groups using the independent samples *t*-test, whereas non-normally distributed variables were analyzed with the Mann–Whitney *U* test. Categorical variables were compared using chi-square or Fisher’s exact tests as appropriate. For the primary binary pain outcome, risk estimates are reported where calculable from the available aggregate data. The multivariable logistic regression is interpreted as exploratory rather than confirmatory. Because participant-level data were not available for this revision, new participant-level ordinal, quantile, Hodges–Lehmann, or fully adjusted sensitivity analyses could not be validly reconstructed. Covariate selection and model interpretation are therefore presented cautiously, and no causal inference is based on the regression model.

## Results

**Fig. 1:**
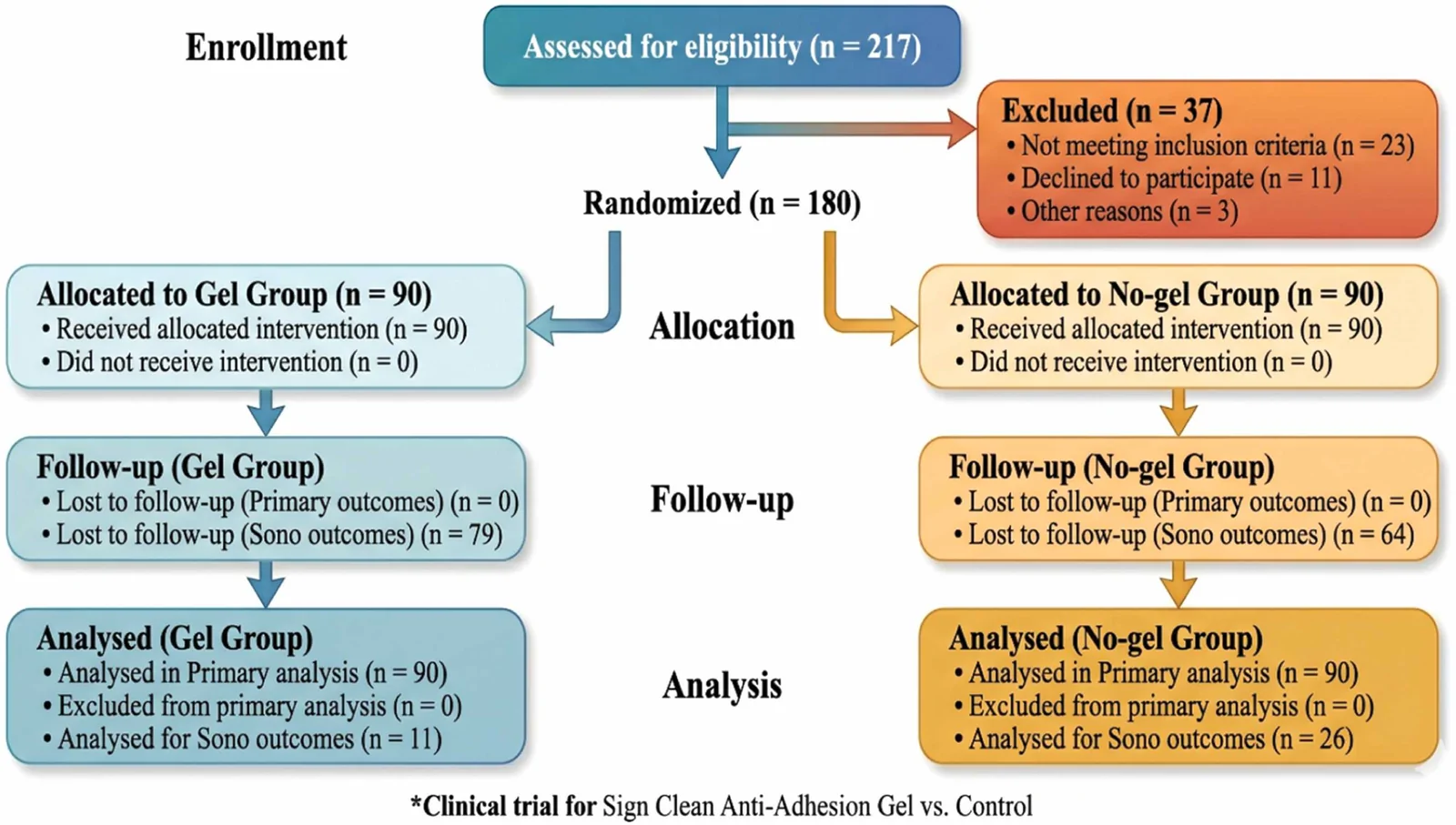
Flow Diagram.

**Fig. 2:**
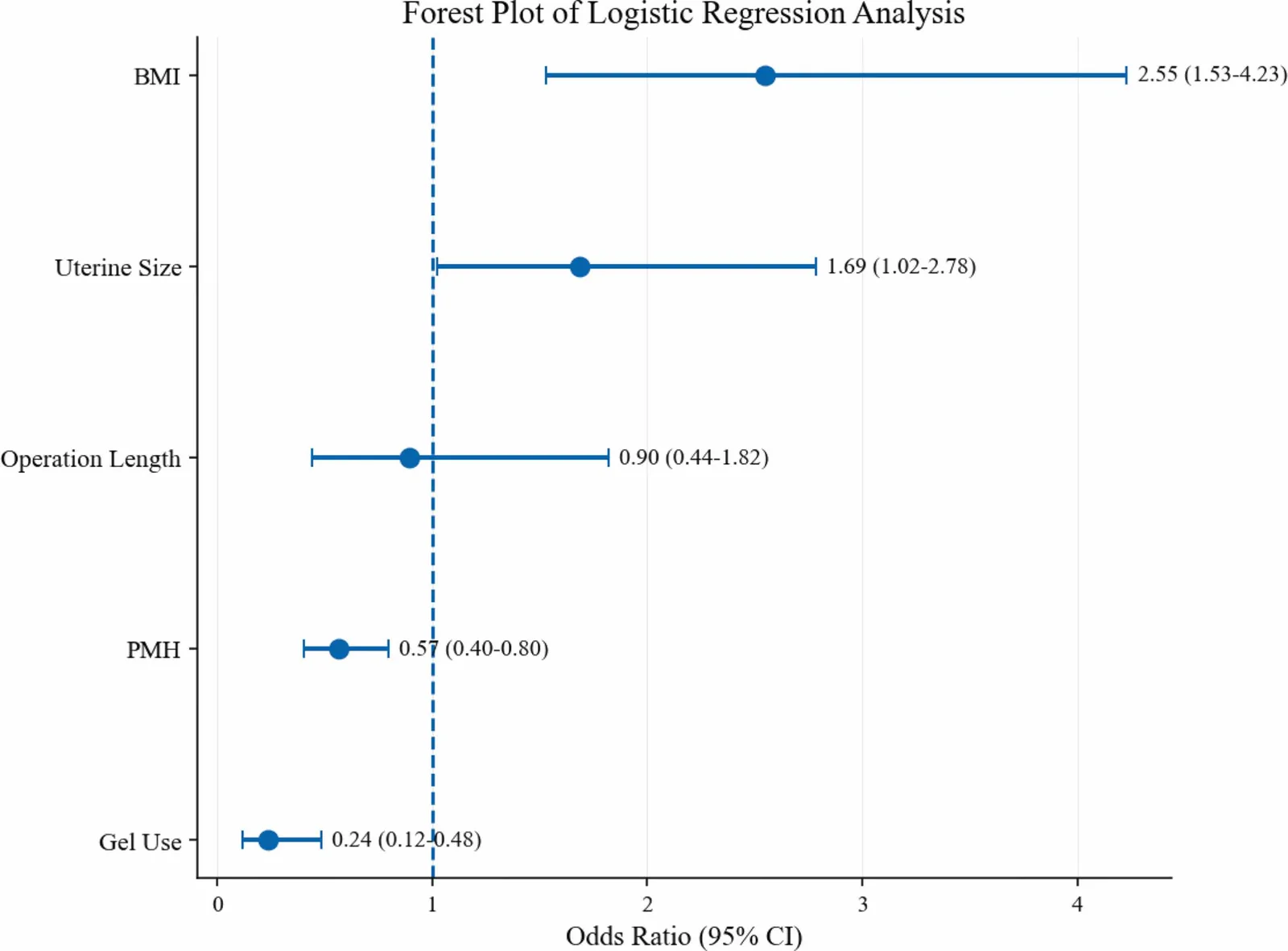
Forest Plot of Logistic Regression.

A total of 217 patients were assessed for eligibility. Of these, 37 were excluded (23 did not meet inclusion criteria, 11 declined to participate, and 3 for other reasons) (see Fig. 1, Fig. 2 and Table 1, Table 2, Table 3, Table 4)

**Fig. 3:**
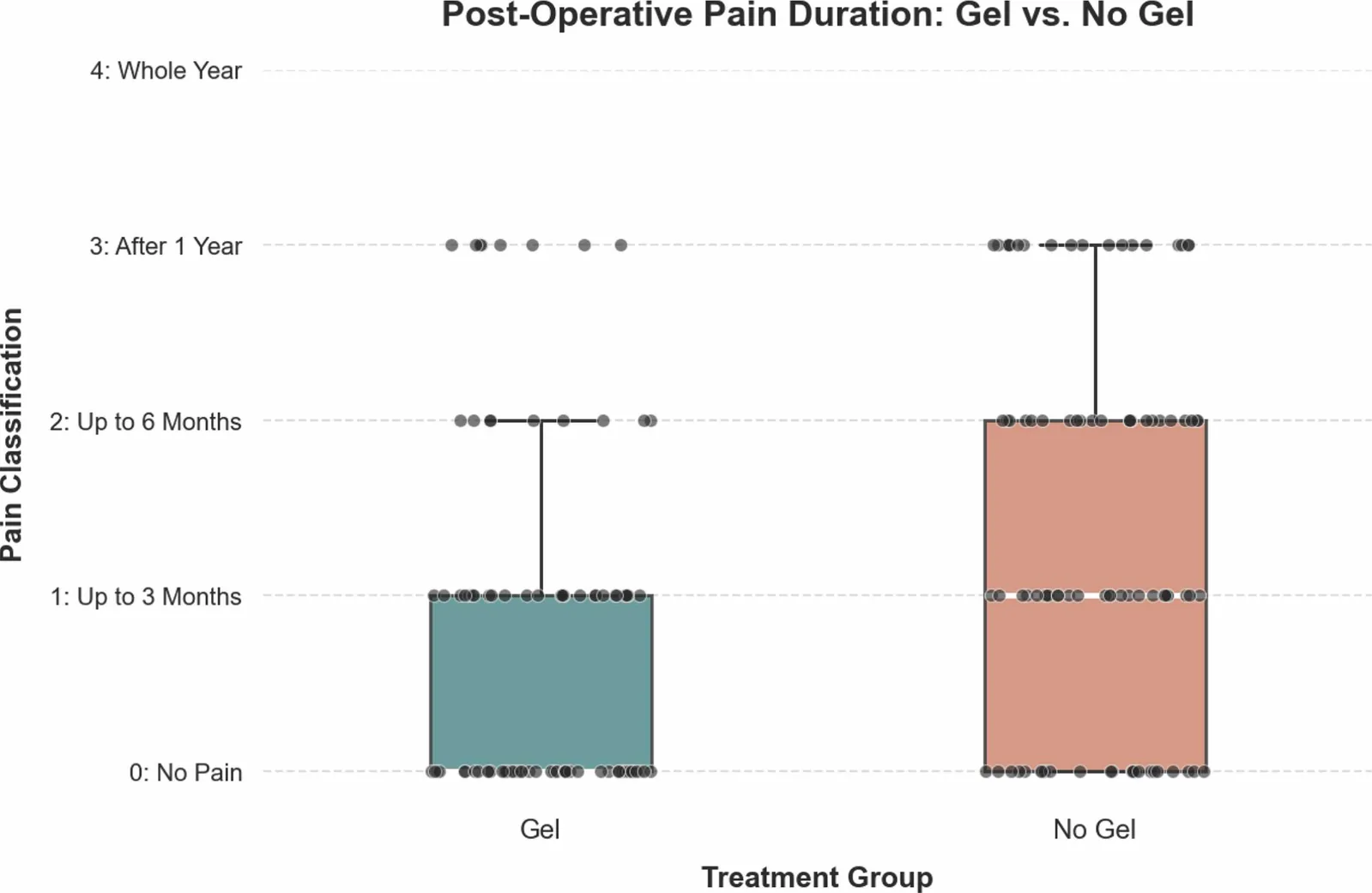
Pain Score Comparison.

**Fig. 4:**
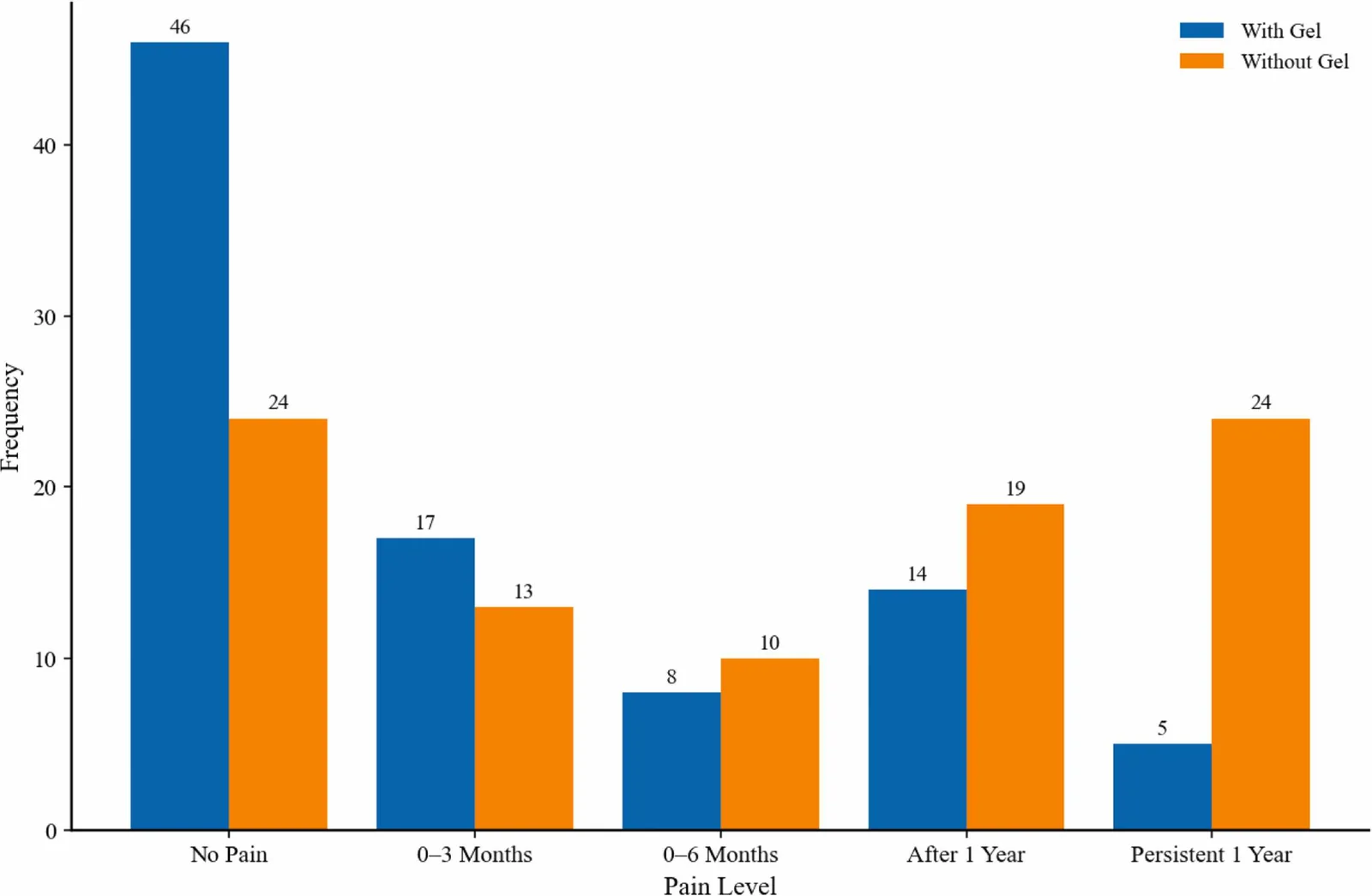
Pain Level Distribution.

**Fig. 5:**
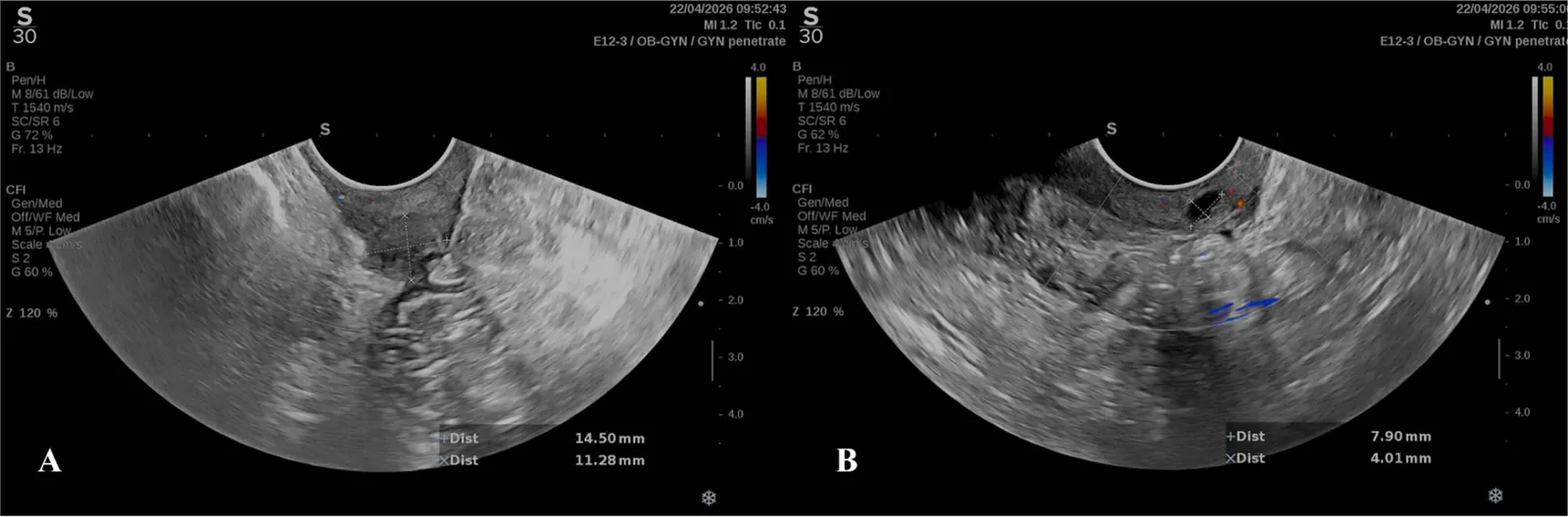
Transvaginal ultrasound images of a 46-year-old woman 12 months after total laparoscopic hysterectomy with ovarian preservation. **(A)** Hypoechoic nodule at the vaginal cuff measuring 14.5 × 11.3 mm (calipers), suggestive of post-surgical changes or small vaginal myoma. **(B)** Thin-walled cyst in the anterior upper vaginal wall measuring 7.9 × 4.0 mm (calipers). Both ovaries are normal in size and echogenicity with preserved mobility. Mild free fluid is present in the pelvic cavity.

**Table 1:** Baseline Characteristics of Study Population.

**Variable**	**With Gel (n = 90)**	**Without Gel (n = 90)**	**P-value**
Age (years), mean ± SD	46.32 ± 3.58	45.69 ± 3.79	0.251
BMI category, n (%)			0.301
- Normal (<25 kg/m²)	37 (41.1)	42 (46.7)	
- Overweight (25–29.9 kg/m²)	31 (34.4)	28 (31.1)	
- Obese (≥30 kg/m²)	22 (24.4)	20 (22.2)	
Past medical history (PMH), any comorbidity, n (%)	29 (32.2)	33 (36.7)	0.429
Indication for hysterectomy, n (%)			0.359
- Abnormal uterine bleeding	58 (64.4)	66 (73.3)	
- Uterine myoma	24 (26.7)	17 (18.9)	
- Adenomyosis / other	8 (8.9)	7 (7.8)	
Uterine size, n (%)			0.042*
- Normal / small	47 (52.2)	60 (66.7)	
- Large (16–24 weeks)	43 (47.8)	30 (33.3)	
Length of operation, n (%)			0.056
- ≤ 120 min	28 (31.1)	37 (41.1)	
- 121–180 min	39 (43.3)	33 (36.7)	
- > 180 min	23 (25.6)	20 (22.2)	
Previous surgical history (PSH), n (%)	48 (53.3)	68 (75.6)	0.005*

**Table 2:** Comparison of Postoperative Pelvic Pain Outcomes Between Study Groups.

**Variable**	**With Gel (n = 90)**	**Without Gel (n = 90)**	**Statistical Test**	**P-value**
Pain score (median, IQR)	0 (0–2)	2 (0–4)	Mann–Whitney U	< 0.001*
Pain level category, n (%)			Chi-square	0.001*
- No pain	46 (51.1%)	24 (26.7%)		
- Resolved within first 3 months	27 (30.0%)	26 (28.9%)		
- Resolved within first 6 months	9 (10.0%)	22 (24.4%)		
- Persistent pain at 12 months	8 (8.9%)	18 (20.0%)		
Need for analgesics, n (%)			Chi-square	0.003*
- No	67 (74.4%)	47 (52.2%)		
- Yes	23 (25.6%)	43 (47.8%)		

Footnote: Pain level categories represent the period during which pain resolved, based on patient recall at the 12-month assessment. Categories are mutually exclusive. Because these categories were based on retrospective recall rather than serial prospectively collected assessments, they may be affected by recall bias. The principal binary pain analysis used NRS ≥ 1; this threshold captures any reported pain and may include mild discomfort.

**Table 3:** Comparison of Postoperative Complications Between Study Groups.

**Variable**	**With Gel (n = 90)**	**Without Gel (n = 90)**	**Statistical Test**	**P-value**
Postoperative complications	11 (12.2%)	18 (20.0%)	Chi-square	0.224
Suspected adhesion-related sonographic findings	3 (3.3%)	16 (17.8%)	Fisher’s exact	0.003*
Cyst formation / cystic lesions	6 (6.7%)	18 (20.0%)	Fisher’s exact	0.015*

**Table 4:** Multivariate Logistic Regression Analysis for Predictors of Chronic Pelvic Pain.

**Variable**	**Category / Unit**	**Adjusted OR**	**95% CI**	**P-value**
Gel use	With gel vs. without gel	0.48	0.26 – 0.88	0.018*
Age	Per year increase	1.02	0.98 – 1.06	0.271
BMI	Overweight vs. normal	1.34	0.72 – 2.50	0.356
	Obese vs. normal	1.89	1.01 – 3.54	0.046*
PMH	DM	1.52	0.71 – 3.25	0.279
	HTN	1.38	0.65 – 2.91	0.398
	Hypothyroidism	1.61	0.74 – 3.48	0.230
Uterine size	Enlarged (16–24 weeks) vs. normal	1.76	0.95 – 3.28	0.072
	≥ 20 weeks vs. normal	2.21	1.12 – 4.38	0.022*
Operation length	180 min vs. 120 min	1.67	0.90 – 3.08	0.104
	240 min vs. 120 min	2.58	1.29 – 5.17	0.007*
Surgical type (PSH)	Yes vs. No	0.91	0.52 – 1.60	0.743

### Baseline characteristics

The two groups were largely comparable at baseline (Table 1). Mean age was 46.32 ± 3.58 years in the gel group and 45.69 ± 3.79 years in the control group (P = 0.251). Distribution of body mass index (BMI) categories and past medical history were also similar between groups (P = 0.301 and P = 0.429, respectively). The most common indication for hysterectomy was abnormal uterine bleeding in both groups (64.4% vs 73.3%), with no significant difference (P = 0.359).

However, two baseline variables differed significantly. Larger uterine sizes (16–24 weeks) were more frequent in the gel group (47.8%) than in the control group (33.3%), while previous surgical history was more common in controls (75.6% vs 53.3%). These opposing imbalances should be interpreted cautiously and do not permit directional inference regarding residual confounding.

Although randomization resulted in some imbalances, particularly in uterine size and previous surgical history, these opposing differences underscore the importance of cautious interpretation and do not by themselves establish residual confounding or strengthen causal inference.

### Postoperative pelvic pain

Patients in the gel group experienced lower patient-reported pelvic pain at 12-month follow-up compared with the control group (Table 2). The median pain score on the Numerical Rating Scale was 0 (IQR: 0–2) in the gel group and 2 (IQR: 0–4) in the control group (P < 0.001). Pain defined as NRS ≥ 1 was reported by 44/90 (48.9%) participants in the gel group versus 66/90 (73.3%) in the control group. The absolute risk difference was −24.4 %age points (95% CI, −38.2 to −10.7), and the relative risk was 0.67 (95% CI, 0.52–0.85). These estimates describe any reported pain and should not be interpreted as evidence of clinically significant chronic postsurgical pain. A participant-level NRS ≥ 3 analysis could not be reconstructed from the available aggregate data.

### Perioperative complications

Postoperative complications were numerically lower in the gel group (12.2% vs 20.0%), but the difference was not statistically significant (P = 0.224). Exploratory sonographic findings suggestive of adhesions were less frequent in the gel group (3.3% vs 17.8%; P = 0.003), but ultrasound cannot objectively establish or grade postoperative adhesions. Cystic lesions were also less frequent in the gel group (6.7% vs 20.0%; P = 0.015); this finding is exploratory and may reflect chance, multiple comparisons, ascertainment differences, baseline ovarian characteristics, or functional cysts unrelated to adhesions.

### Predictors of chronic pelvic pain

Multivariable logistic regression identified factors associated with the NRS ≥ 1 pain outcome (Table 4). Gel use was associated with lower odds of reported pelvic pain (adjusted OR 0.48, 95% CI 0.26–0.88; P = 0.018). This model should be considered exploratory. Odds ratios are reported because they were generated by the original logistic model, but they are not interpreted as equivalent to risk ratios. The model does not establish causality, and the absence of participant-level data precludes independent reconstruction of event-per-parameter diagnostics or alternative adjusted models in this revision.

## Discussion

This secondary analysis of a patient- and outcome-assessor-blinded randomized controlled trial found that intraoperative application of Singclean hyaluronic acid-based gel was associated with lower patient-reported pelvic pain at 12 months after ovarian-preserving laparoscopic hysterectomy. The principal finding is patient-reported pain; the study does not establish that the effect was mediated by reduced adhesions. The parent trial was designed around a different sample-size basis and was not specifically powered for long-term pain, and quantitative baseline pelvic pain was not available.

Our findings are biologically consistent with contemporary evidence that hyaluronic-acid barriers can reduce postoperative adhesion formation in gynecologic surgery. Recent systematic-review evidence has reported reductions in moderate-to-severe adhesions and in the number of adhesion sites with hyaluronan products. These findings support the anti-adhesive rationale for the intervention, but anatomical adhesion prevention should not be assumed to translate directly into less chronic postsurgical pain.

Most previous studies have focused on short-term adhesion scores assessed at second-look laparoscopy rather than patient-centered outcomes such as pain at one year. Contemporary evidence therefore supports an adhesion-prevention effect more strongly than a pain-prevention effect. The present analysis adds hypothesis-generating long-term patient-reported data, but it cannot establish that reduced adhesion formation caused pain relief. Persistent pain after hysterectomy is multifactorial and may involve preoperative pain, central sensitization or nociplastic mechanisms, psychological distress, acute postoperative pain, inflammatory or neuropathic processes, and perioperative factors.

The lower pelvic pain observed in the gel group is compatible with the hypothesis that postoperative adhesion-related changes may contribute to pain in some patients, particularly when ovaries are preserved, but the present study cannot confirm this mechanism. Adhesions were assessed indirectly by ultrasound, and the relationship between sonographic findings and true adhesion burden is imperfect. The cyst finding is exploratory and should not be used as evidence of an anti-adhesive mechanism. The opposing baseline imbalances in uterine size and previous surgical history underscore the need for cautious interpretation rather than permitting directional inference regarding residual confounding. The multivariable analysis is exploratory and does not establish an independent causal effect of gel application.

Strengths of this study include its randomized patient- and outcome-assessor-blinded design, complete 12-month pain follow-up, intention-to-treat analysis, and patient-centered outcome. Important limitations include the use of NRS ≥ 1 as the principal binary threshold, which may capture mild discomfort; absence of quantitative preoperative pain assessment; retrospective recall of pain over the preceding year; assessment at a single 12-month time point rather than serial predefined intervals; inability to reconstruct an NRS ≥ 3 analysis from the available aggregate data; potential post hoc/secondary-analysis bias; multiple comparisons among exploratory outcomes; absence of a placebo gel; inability to account for surgeon-level clustering in a participant-level model; incomplete sonographic follow-up; absence of detailed perioperative analgesic exposure; incomplete verification of the original trial endpoint hierarchy and statistical-plan timing; single-center design; and limited generalizability. Ultrasound cannot objectively diagnose or grade adhesions, and the imaging findings therefore remain exploratory. Missing imaging data may also introduce attrition bias. These limitations mean that the observed association should not be interpreted as definitive evidence of chronic postsurgical pain prevention.

The present findings suggest that intraoperative hyaluronic acid gel may be associated with lower patient-reported pelvic pain after ovarian-preserving laparoscopic hysterectomy, but they do not establish routine clinical effectiveness or a causal adhesion-mediated mechanism. Future multicentre randomized trials should prospectively specify clinically meaningful pain thresholds, serial pain trajectories, analgesic use, functional interference, quality of life, and validated chronic postsurgical pain criteria, with adequate power for patient-reported outcomes and prespecified mechanistic and imaging analyses.

## Conclusion

In this secondary analysis of a randomized, patient- and outcome-assessor-blinded trial, intraoperative hyaluronic acid gel application during ovarian-preserving laparoscopic hysterectomy was associated with lower patient-reported pelvic pain at 12 months. Although the magnitude of the observed difference is clinically promising, the principal binary threshold was NRS ≥ 1, baseline pain trajectories were not prospectively quantified, and the parent trial was not powered specifically for long-term pain. Exploratory ultrasound findings cannot establish the presence or absence of adhesions or a causal mechanism. These findings justify a larger multicentre randomized trial with prospectively specified patient-reported pain, functional, and quality-of-life outcomes.

## CRediT authorship contribution statement

**Maryam Hashemi:** Project administration, Methodology, Investigation, Funding acquisition, Formal analysis, Data curation, Conceptualization. **Fatemeh Radmanesh:** Validation, Supervision, Software, Resources, Project administration, Methodology. **Safoura Rouholamin:** Writing – original draft, Visualization, Validation, Supervision, Software, Resources. **Mohammadreza Elhaie:** Writing – review & editing, Writing – original draft, Visualization, Validation, Supervision, Software.

## Ethical approval

The study protocol was approved by the Ethics Committee of Blinded University of Medical Sciences (BLINDED).

## Ethical Approval

The study was approved by the Ethics Committee of Blinded University of Medical Sciences (approval number: BLINDED). All procedures performed in this study were in accordance with the ethical standards of the institutional research committee and with the 1964 Helsinki Declaration and its later amendments.

## Ethical Approval

The study was approved by the Ethics Committee of Isfahan University of Medical Sciences (approval number: IR.MUI.MED.REC1404.042). All procedures performed in this study were in accordance with the ethical standards of the institutional research committee and with the 1964 Helsinki Declaration and its later amendments.

## Ethical approval

The study protocol was approved by the Ethics Committee of Isfahan University of Medical Sciences (IR.MUI.MED.REC1404.042). The trial was registered in the Iranian Registry of Clinical Trials (IRCT20230102057020N1).

## Informed Consent

Written informed consent was obtained from all individual participants included in the study prior to enrollment.

## Funding

This study was supported by a grant from Blinded University of Medical Sciences, Blinded, Blinded (Grant No. 3403390). The funder had no role in study design, data collection, data analysis, data interpretation, or writing of the report.

## Funding

This study was supported by a grant from 10.13039/501100003970Isfahan University of Medical Sciences, Isfahan, Iran (Grant No. 3403390).

## Declaration of Competing Interest

The authors declare that they have no known competing financial interests or personal relationships that could have appeared to influence the work reported in this paper.

## Data Availability

The datasets generated and/or analyzed during the current study are available from the corresponding author on reasonable request.
